# Cerebral tau pathology in cerebral amyloid angiopathy

**DOI:** 10.1093/braincomms/fcae086

**Published:** 2024-03-12

**Authors:** Hsin-Hsi Tsai, Chia-Ju Liu, Bo-Ching Lee, Ya-Fang Chen, Ruoh-Fang Yen, Jiann-Shing Jeng, Li-Kai Tsai

**Affiliations:** Department of Neurology, National Taiwan University Hospital, Taipei 100225, Taiwan; Department of Nuclear Medicine, National Taiwan University Hospital, Taipei 100225, Taiwan; Department of Medical Imaging, National Taiwan University Hospital, Taipei 100225, Taiwan; Department of Medical Imaging, National Taiwan University Hospital, Taipei 100225, Taiwan; Department of Nuclear Medicine, National Taiwan University Hospital, Taipei 100225, Taiwan; Department of Neurology, National Taiwan University Hospital, Taipei 100225, Taiwan; Department of Neurology, National Taiwan University Hospital, Taipei 100225, Taiwan

**Keywords:** cerebral amyloid angiopathy, small vessel disease, intracerebral haemorrhage, Alzheimer’s disease, cortical superficial siderosis

## Abstract

Tau, a hallmark of Alzheimer’s disease, is poorly characterized in cerebral amyloid angiopathy. We aimed to assess the clinico-radiological correlations between tau positron emission tomography scans and cerebral amyloid angiopathy. We assessed cerebral amyloid and hyperphosphorylated tau in patients with probable cerebral amyloid angiopathy (*n* = 31) and hypertensive small vessel disease (*n* = 27) using ^11^C-Pittsburgh compound B and ^18^F-T807 positron emission tomography. Multivariable regression models were employed to assess radio-clinical features related to cerebral tau pathology in cerebral amyloid angiopathy. Cerebral amyloid angiopathy exhibited a higher cerebral tau burden in the inferior temporal lobe [1.25 (1.17–1.42) versus 1.08 (1.05–1.22), *P* < 0.001] and all Braak stage regions of interest (*P* < 0.05) than hypertensive small vessel disease, although the differences were attenuated after age adjustment. Cerebral tau pathology was significantly associated with cerebral amyloid angiopathy-related vascular markers, including cortical superficial siderosis (*β* = 0.12, 95% confidence interval 0.04–0.21) and cerebral amyloid angiopathy score (*β* = 0.12, 95% confidence interval 0.03–0.21) after adjustment for age, ApoE4 status and whole cortex amyloid load. Tau pathology correlated significantly with cognitive score (Spearman’s *ρ*=−0.56, *P* = 0.001) and hippocampal volume (−0.49, *P* = 0.007), even after adjustment. In conclusion, tau pathology is more frequent in sporadic cerebral amyloid angiopathy than in hypertensive small vessel disease. Cerebral amyloid angiopathy-related vascular pathologies, especially cortical superficial siderosis, are potential markers of cerebral tau pathology suggestive of concomitant Alzheimer’s disease.

## Teaser

In this study performed by Tsai *et al.*, cerebral tau burden measured on positron emission tomography is higher in cerebral amyloid angiopathy than in hypertensive small vessel disease. Cerebral amyloid angiopathy-related vascular pathologies, especially cortical superficial siderosis, are potential markers of cerebral tau pathology suggestive of concomitant Alzheimer’s disease.

## Graphical Abstract

**Graphical Abstract:**
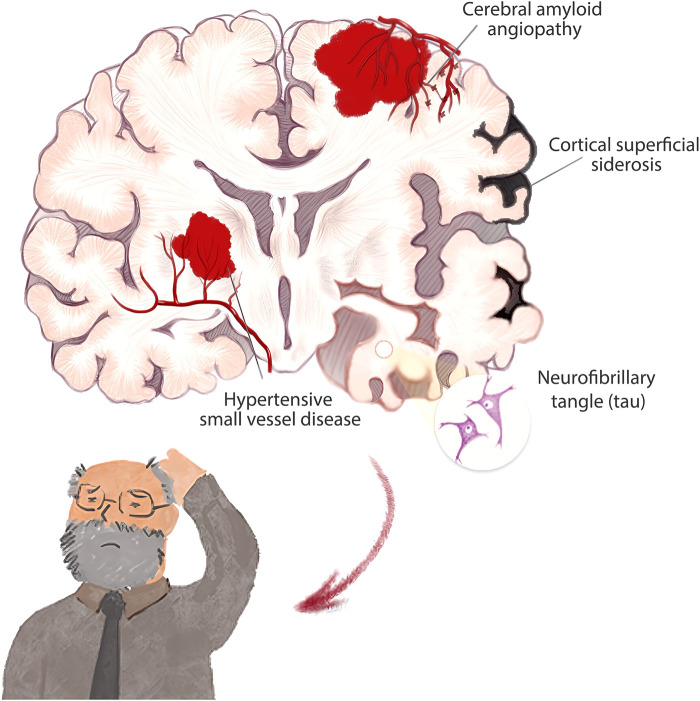

## Introduction

Cerebral amyloid angiopathy (CAA), which involves cerebrovascular deposition of β-amyloid (Aβ) proteins, is the major vascular aetiology underlying spontaneous lobar intracerebral haemorrhage (ICH) in elderly individuals.^1^ Aβ plaques in the parenchyma and neurofibrillary tangles comprised of hyperphosphorylated tau proteins are the histopathological hallmarks of Alzheimer’s disease. The shared role of Aβ in both CAA and Alzheimer’s disease results in an overlap between these age-related disorders.^2,3^ Both CAA and Alzheimer’s disease ultimately lead to neurodegeneration, although probably via different mechanisms. Vascular dysfunction is considered to be a major pathway for the cortical atrophy in CAA,^4^ while the role of concomitant tau pathology in CAA is still undetermined.

Tau pathology has only rarely been investigated in CAA; one cohort study from a memory clinic showed that the presence of tau in CAA potentially influences cognition.^5^ Determination of the accumulation of tau in CAA is challenging, since there are no specific MRI markers that can directly measure tau pathology. Studies investigating CSF biomarkers have shown a marginal increase in CSF p-tau levels in patients with sporadic CAA compared with healthy controls, suggesting either lower amounts of tau are deposited (compared with patients with Alzheimer’s disease) or heterogeneous accumulation of tau in patients with CAA.^6^ However, it remains unclear whether the presence of intracerebral tau pathology in sporadic CAA is suggestive of coexisting Alzheimer’s disease, as there is also evidence of perivascular tau neurites around amyloid-laden vessels.^7-9^ Tau PET imaging can be used to detect the accumulation and distribution of intracellular tau protein *in vivo*^10^; thus, this imaging modality could help to delineate whether the tau pathology in CAA is similar to that of Alzheimer’s disease and explore how tau correlates with the clinical and neuroimaging manifestations of CAA.

Survivors of spontaneous ICH are presumed to have the vascular aetiology of cerebral small vessel disease (SVD). This intrinsic microangiopathy not only leads to the rupture of small vessels but also results in progressive cognitive dysfunction.^11,12^ CAA is one of the most common forms of sporadic SVD in ICH survivors. Thus, concomitant tau pathology is expected in a subset of patients who have suffered ICH, which may predispose these patients to develop cognitive impairment and neurodegeneration. In this cross-sectional study, we aimed to determine whether there is a higher frequency of Alzheimer’s disease-related tau pathology in CAA compared with other forms of sporadic SVD. We included patients who had survived ICH with a presumed vascular aetiology of either CAA or hypertensive SVD (HTN-SVD). We compared the tau burdens in Alzheimer’s disease-signature regions between these two groups using PET imaging. Furthermore, we investigated the clinical and radiological features associated with cerebral tau accumulation in the patients with CAA. As a secondary objective, we tested our hypothesis that tau pathology independently contributes to neurodegeneration in CAA.

## Materials and methods

### Patient enrolment

**Figure 1:**
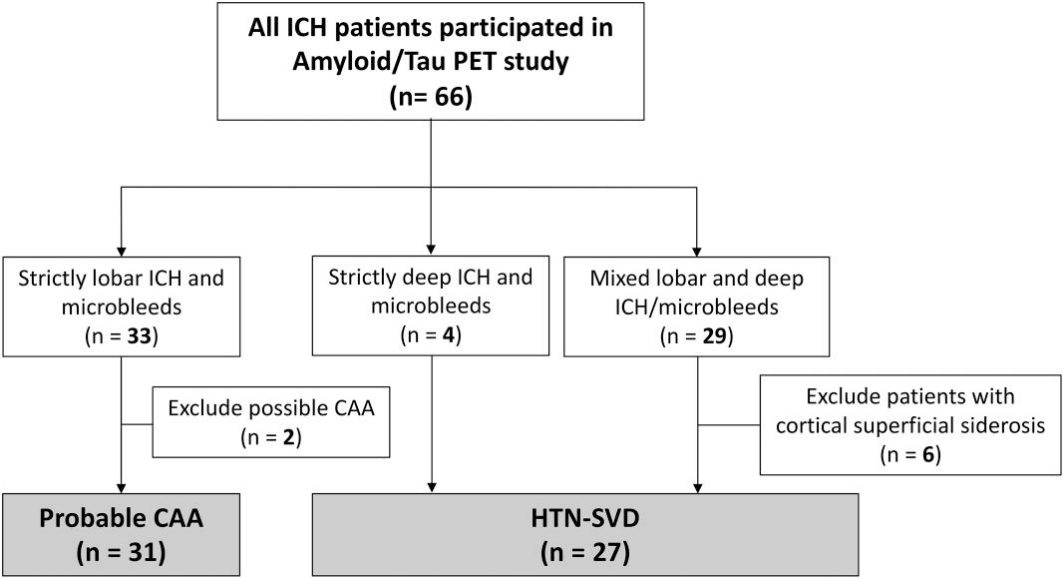
**Flowchart of patient enrolment.** Sixty-six patients with SVD-related spontaneous ICH were recruited to participate in this study; 31 patients with probable CAA and 27 patients with HTN-SVD (four patients with strictly deep ICH and microbleeds; 23 patients with mixed lobar and deep ICH and microbleeds) were included in the final analysis.

For this cross-sectional study, we prospectively enrolled patients who had survived symptomatic spontaneous ICH (i.e. patients with intrinsic SVD) from February 2019 onwards. The flowchart of patient enrolment is shown in Fig. 1. All patients were recruited in the chronic phase (defined as at least 6 months after the index ICH event) and underwent comprehensive neuroimaging studies including MRI and PET. According to the Boston criteria 2.0,^13^ patients with lobar ICH(s) involving the cerebral cortex and underlying white matter with strictly lobar cerebral microbleeds (CMBs), cortical superficial siderosis (cSS) or white matter features [severe perivascular spaces (PVSs) in the centrum semiovale (CSO) or white matter hyperintensities (WMHs) in a multi-spot pattern] were defined as having possible (*n* = 2) or probable CAA (*n* = 31). For the purpose of this study, only patients whose MRI findings met the criteria of probable CAA as per the Boston criteria 2.0 were included in further analyses. Patients with haemorrhage/CMBs exclusively located in deep brain regions (basal ganglia, thalamus or pons; *n* = 4) or patients with a combination of mixed lobar and deep bleeds (*n* = 29) were categorized as HTN-SVD, as these haemorrhages are probably caused by hypertensive microangiopathy.^14,15^ In the HTN-SVD group, we then excluded patients with the presence of cSS (*n* = 6), as convincing evidence suggests cSS is associated with underlying concomitant CAA.^15,16^

Baseline clinical data were collected by the investigators through a comprehensive review of medical records and interviewing each participant. We collected data on the following demographic characteristics: age, sex, years of education, ApoE4 status and diagnosis of chronic hypertension, diabetes and hypercholesterolaemia. At enrolment, the cognitive status of each patient was assessed through a combination of history taking and an objective cognitive assessment that included the Mini-Mental State Examination (MMSE) and Clinical Dementia Rating score.

### MRI acquisition and analysis

Brain MRIs were obtained using a 3-T scanner (Siemens Verio, TIM or mMR, Siemens Medical Solutions, Malvern, PA, USA). The imaging protocols included a T_1_-weighted magnetization-prepared rapid gradient-echo imaging (MPRAGE, flip angle 9, repetition time/echo time = 1460/2.39 ms, field of view = 25.6 cm and slice thickness = 1 mm), T_2_-weighted imaging (repetition time/echo time = 3530/83 ms, field of view = 23 cm and slice thickness = 5 mm), FLAIR (repetition time/echo time = 10 000/89 ms, field of view = 23 cm and slice thickness = 5 mm), susceptibility-weighted imaging (flip angle 15, repetition time/echo time = 28/20 ms, matrix number = 221 × 320, field of view = 23 cm and slice thickness = 2 mm), diffusion-weighted imaging and apparent diffusion coefficient maps, as previously described.^17^

MRI markers related to cerebral SVD were evaluated based on the Standards for Reporting Vascular Changes on Neuroimaging criteria (STRIVE2).^18^ In addition, 30 scans were randomly selected for independent assessment by two raters (author H.H.T. and B.C.L.) to determine the inter-rater agreement of the visually assessed MRI markers. The inter-rater agreement was good for detection of lobar CMB (*k*, 0.71, 95% CI 0.35–1.00), deep CMB (*k*, 1.00, 95% CI 1.00–1.00), cSS (*k*, 0.71, 95% CI 0.46–0.97), high-degree PVS in CSO (*k*, 0.73, 95% CI 0.49–0.97), high-degree PVS in basal ganglia (*k*, 0.73, 95% CI 0.48–0.98) and lacune (*k*, 0.83, 95% CI, 0.60–1.00).

The number of CMBs and the presence of cSS were evaluated using axial susceptibility-weighted imaging sequences.^19,20^ CMBs were defined as lesions with homogeneous round signal loss of less than 10 mm on susceptibility-weighted imaging, but not symmetric hypointensities and flow voids from blood vessels. The number of CMBs in the lobar regions (i.e. the frontal, temporal, parietal, occipital and insular cortices), deep regions (i.e. the brainstem, basal ganglia,^21^ thalamus, internal capsule, external capsule, corpus callosum and deep periventricular white matter) and cerebellum was counted. The total cSS multi-focality score was determined using a previously proposed 5-point (range, 0–4) scoring system^20^ that assesses both the multi-focality and the extensiveness of cSS. Lacunes were evaluated in the supratentorial region and defined as ‘round or ovoid, subcortical, fluid-filled cavities, from 3 to 15 mm in diameter’.^22,23^ WMH volume was calculated based on fluid-attenuated inversion recovery imaging of the ICH-free hemisphere and multiplied by 2, as we previously proposed.^24^ MRI-visible PVSs were evaluated on T_2_-weighted imaging and defined as sharply delineated structures measuring <3 mm following the course of perforating or medullary vessels.^25^ High-degree PVS was defined as >20 visible PVS in the CSO or in the basal ganglia on the side of the brain with more severe involvement.^25,26^ CAA score (range, 0–6) was calculated based on a previously proposed scale that assesses CAA-related SVD markers (lobar CMB, cSS, CSO-PVS and WMH).^27^ All MRI scans were also processed using FreeSurfer software v6.0.0 (http://surfer.nmr.mgh.harvard.edu/) to determine the mean cerebral cortex thickness and hippocampal volume using a T_1_-weighted MPRAGE sequence.

### PET acquisition and analysis

^11^C-Pittsburgh compound B (PiB) and ^18^F-T807 were prepared at the Cyclotron and Radiopharmaceutical Laboratory of National Taiwan University Hospital. PET images (Discovery ST, GE Healthcare) were acquired over 30 min approximately 40 min after injection of 10 mCi ^11^C-PiB or ^18^F-T807. As we previously described,^17^ PET data were reconstructed via ordered set expectation maximization and corrected for attenuation. The PET data in the ICH-free hemisphere were semi-quantitatively analysed and expressed as the standardized uptake value ratios (SUVRs) of the regions of interest (ROIs) using PMOD software. The PiB-PET scans were assessed in each lobe (frontal, temporal, parietal and occipital lobes) and in the whole cerebral cortex using the cerebellar cortex as the reference region. The T807 PET scans were assessed to determine the SUVRs for the inferior temporal lobe and Braak stage ROIs [Braak Stages I/II (transentorhinal), III/IV (limbic) and V/VI (neocortical)].^17,28^ We defined tau PET positivity based on a cut-off value of a SUVR ≥ 1.3 in any of the ROIs, according to a previous report.^29^

All neuroimaging studies (MRI and PET scans) were performed within 3 months of enrolment of each participant.

### Statistical analysis

We compared the baseline demographic information and neuroimaging variables for the patients with CAA and HTN-SVD. Discrete variables are presented as counts (%) and continuous variables are presented as mean (± standard deviation) or median (interquartile range), as appropriate, based on their distribution. Between-group comparisons (CAA versus HTN-SVD) of mean (e.g. age and MMSE), median (e.g. amyloid and tau PET findings) and proportions were performed using the independent sample *t*-test, Mann–Whitney *U*-test and Fisher’s exact test, respectively. The median amyloid and tau PET values in the CAA and HTN-SVD groups were further adjusted for age using non-parametric quantile regression analysis.

Within the patients with CAA, in order to explore the correlation between cerebral tau pathology and other neuroimaging signatures, we chose the inferior temporal T807 SUVR as the marker for tau pathology, as this region is considered to be a sensitive ROI for Alzheimer’s disease-related tau accumulation.^10^ All independent variables (neuroimaging signatures) were standardized in the quantile regression models to investigate the association between cerebral tau pathology and vascular markers. Based on the analyses described above, we plotted the receiver operating characteristic curves and used the Youden index to determine the best cut-off values for the presence/absence of cSS, total cSS score and CAA score for the prediction of positive tau scans. The diagnostic value, including sensitivity, specificity, positive predictive value, negative predictive value and the area under the curve (AUC), was determined for each of these parameters. Spearman’s correlation analysis was applied to investigate the relationship between T807 SUVR and neurodegeneration markers (MMSE score, mean hippocampal volume and mean cortical thickness). Quantile regression analyses were then applied to adjust for potential confounders, including age, years of education and CAA scores.

All statistical analyses were performed using SPSS version 25 (SPSS Inc., Chicago, IL, USA) and Stata version 14 (StataCorp LLC, College Station, TX, USA). All tests of significance were two-tailed and based on a threshold for significance of *P* < 0.05. No adjustment for multiple testing was performed in this study.

### Standard protocol approvals, registrations and patient consent

This study was performed with the approval of the institutional review board (201903069RINB) of the National Taiwan University Hospital and in accordance with their guidelines. Written informed consent was obtained from all participants or their family members.

## Results

### Demographics of amyloid and tau burden in CAA versus HTN-SVD

We included 31 patients with probable CAA (mean age, 72.9 ± 7.5 years; 45.2% male) and 27 patients with HTN-SVD (mean age, 67.3 ± 10.0 years; 66.7% male) in this study (Fig. 1). The baseline demographics of these groups are compared in Table 1. Patients with CAA were older (*P* = 0.014) but had similar cognitive performance as patients with HTN-SVD (MMSE score, 21.8 ± 8.7 versus 23.2 ± 8.1, *P* = 0.712). The frequency of the ApoE4 allele was not significantly different between groups (26.7% versus 18.5%, *P* = 0.538). On conventional neuroimaging analysis, the patients with CAA had fewer deep CMBs (0 ± 0 versus 5.1 ± 6.2, *P* < 0.001), fewer lacunes (25.8% versus 59.3%, *P* = 0.016), a higher prevalence of high-degree CSO-PVS (64.5% versus 33.3%, *P* = 0.034) and more cSS (45.2% versus 0%, *P* < 0.001) than patients with HTN-SVD. The number of lobar CMBs, number of cerebellar CMBs, prevalence of high-degree basal ganglia PVS, mean cortical thickness and hippocampal volume were not significantly different between the two groups. As expected, the summary CAA score was significantly higher in patients with CAA than in patients with HTN-SVD (3.3 ± 1.7 versus 1.0 ± 0.6, *P* < 0.001).

**Table 1:** Comparison of demographics in CAA and HTN-SVD

	CAA (*n* = 31)	HTN-SVD (*n* = 27)	*P*-value
Male, %	14 (45.2%)	18 (66.7%)	0.119
Age, y	72.9 ± 7.5	67.3 ± 10.0	0.014
Hypertension, %	22 (71.0%)	21 (77.8%)	0.765
Diabetes, %	7 (22.6%)	8 (29.6%)	0.564
Hyperlipidaemia, %	7 (22.6%)	13 (48.1%)	0.055
MMSE	21.8 ± 8.7	23.2 ± 8.1	0.712
ApoE4^a^ carrier, %	8 (26.7%)	5 (18.5%)	0.538
Cerebral microbleeds			
Lobar CMBs	14.9 ± 30.1	11.9 ± 20.4	0.616
Deep CMBs	0 ± 0	5.1 ± 6.3	<0.001
Cerebellar CMB	3.5 ± 7.8	2.4 ± 9.6	0.265
WMH volume, mL (IQR)	8.4 (3.8–15.0)	13.6 (6.4–19.5)	0.165
Lacunes, %	8 (25.8%)	16 (59.3%)	0.016
MRI-visible enlarged perivascular spaces			
Basal ganglia (>20), %	11 (35.5%)	17 (63.0%)	0.064
Centrum semiovale (>20), %	20 (64.5%)	9 (33.3%)	0.034
Cortical superficial siderosis, %	14 (45.2%)	0 (0%)	<0.001
CAA score	3.3 ± 1.7	1.0 ± 0.6	<0.001
Hippocampal volume, mm^3^	3593 ± 852	3563 ± 484	0.539
Mean cortical thickness, mm	2.29 ± 0.16	2.29 ± 0.19	0.724

Values are mean (± standard deviation), median (IQR) or number (percentage).

CAA, cerebral amyloid angiopathy, CMB, cerebral microbleeds; cSS, cortical superficial siderosis; MMSE, Mini-Mental Status Exam; IQR, interquartile range; WMH, white matter hyperintensities.

^a^*ApoE* genotyping data were not available for one CAA patient.

### Comparison of amyloid and tau burden in CAA versus HTN-SVD

**Figure 2:**
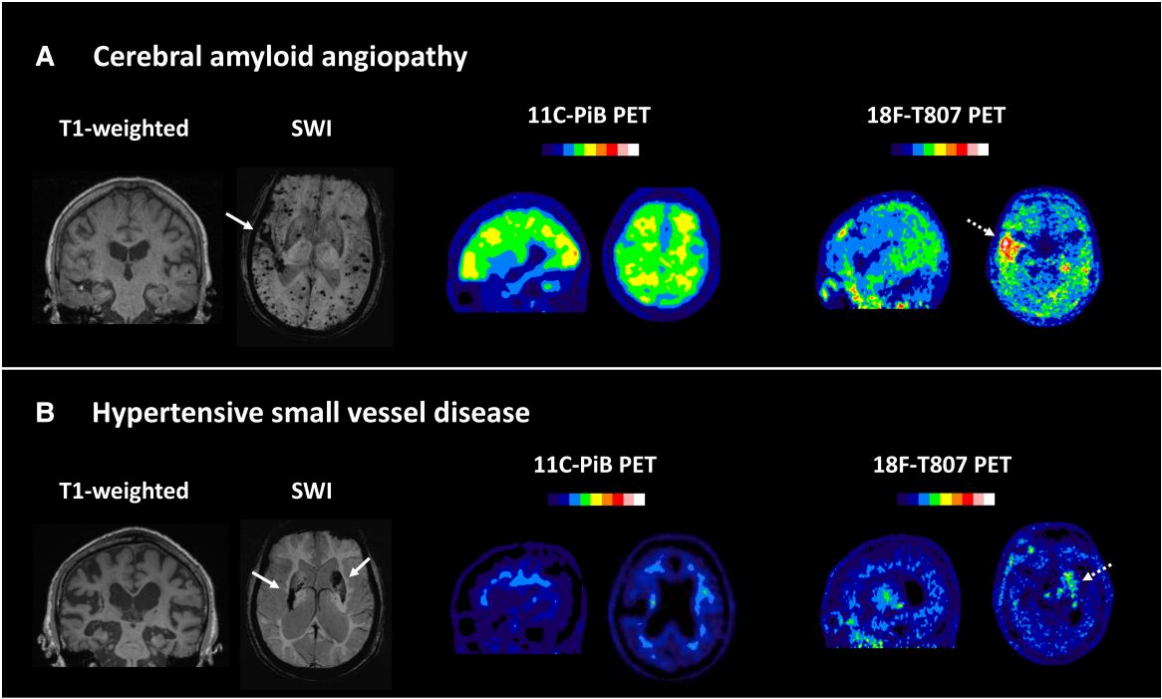
**Representative amyloid and tau scans of CAA and HTN-SVD.** Representative MRI, PiB PET and tau PET images of (**A**) CAA and (**B**) HTN-SVD. Patients with CAA exhibited higher amyloid and tau uptakes compared with patients with HTN-SVD. Off-target binding to haematoma (arrows and dashed arrows) was observed on T807 PET scans.

**Figure 3:**
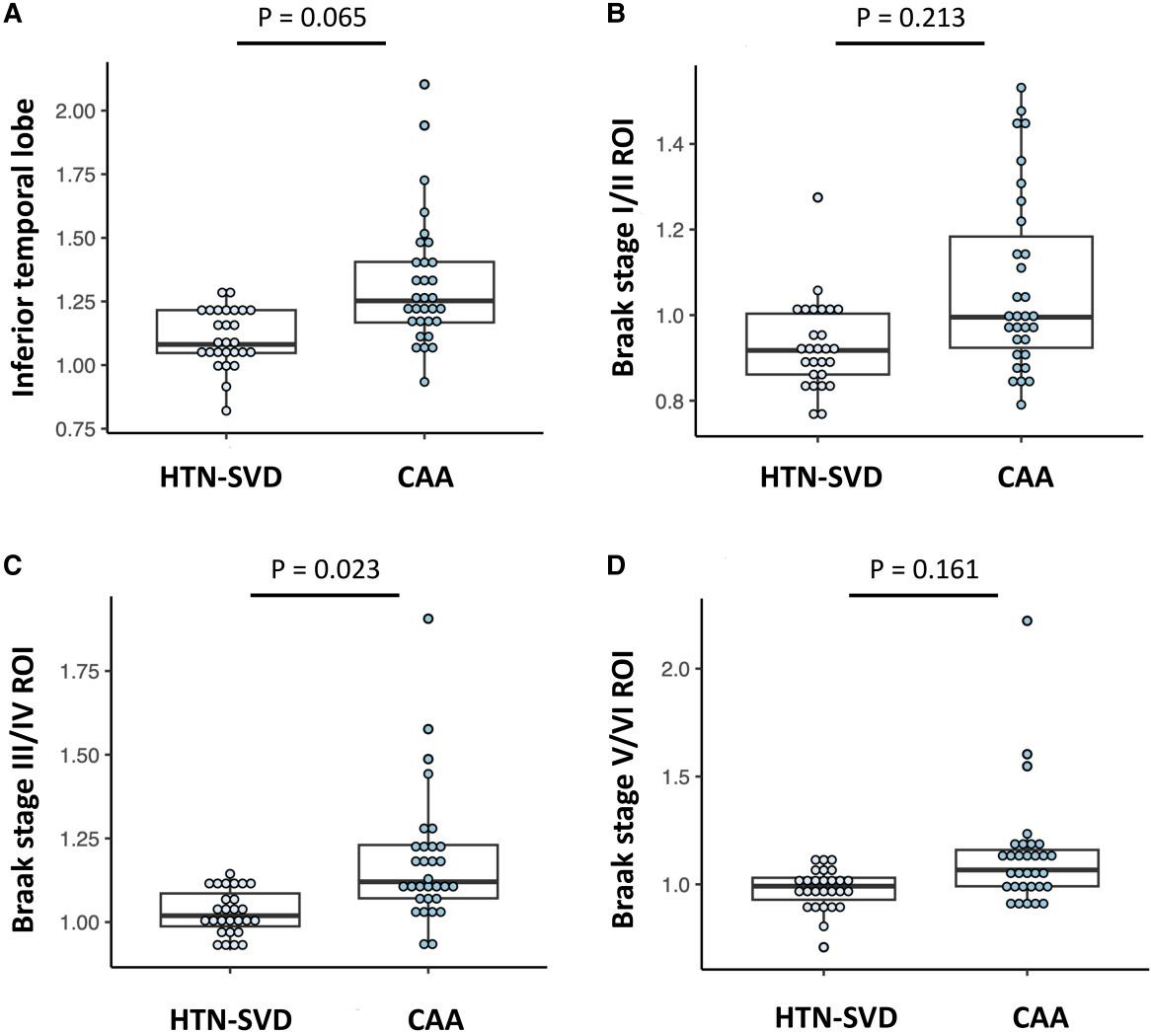
**Comparison of tau uptake between CAA and HTN-SVD.** Box plots showing the trend towards a higher tau burden in CAA (*n* = 31) than HTN-SVD (*n* = 27) in (**A**) the inferior temporal lobe [1.25 (1.17–1.42) versus 1.08 (1.05–1.22), age-adjusted *t* = 1.88, *P* = 0.069], (**B**) Braak stage I/II ROIs [1.00 (0.91–1.22) versus 0.92 (0.85–1.00), age-adjusted *t* = 1.26, *P* = 0.213], (**C**) Braak stage III/IV ROIs [1.12 (1.06–1.24) versus 1.02 (0.99–1.10), age-adjusted *t* = 2.33, *P* = 0.023] and (**D**) Braak stage V/VI ROIs [1.07 (0.99–1.16) versus 0.99 (0.91–1.04), age-adjusted *t* = 1.42, *P* = 0.161].

We next compared the cerebral amyloid and tau burden between the patients with CAA and HTN-SVD using PET scans (Table 2). Figure 2 shows representative neuroimaging of CAA and HTN-SVD. Patients with CAA had higher whole cortex [median SUVR = 1.25 (1.11–1.65) versus 1.08 (1.00–1.14), *P* < 0.001) and regional amyloid burdens (all *P* < 0.001) than patients with HTN-SVD. On tau PET scans, higher tracer uptake was observed in the inferior temporal lobes [1.25 (1.17–1.42) versus 1.08 (1.05–1.22), *P* < 0.001) and all Braak stage ROIs in patients with CAA compared with patients with HTN-SVD (all *P* < 0.05; Table 2). The higher tau burden in CAA remained significant after adjustment for age in Braak III/IV ROIs (*P* = 0.023) and retained a borderline trend in the inferior temporal lobe (*P* = 0.065). Figure 3 displays the box plots of T807 uptake in each group, showing that more extensive tau pathology was detected in CAA compared with HTN-SVD.

**Table 2:** Comparison of amyloid and tau PET findings between CAA and HTN-SVD

Parameter	CAA (*n* = 31)	HTN-SVD (*n* = 27)	*P*-value	Age-adjusted *P*-value
^11^C-PiB PET				
Whole cortex SUVR	1.52 (1.11–1.65)	1.08 (1.00–1.14)	<0.001	<0.001
Frontal SUVR	1.57 (1.09–1.66)	1.04 (0.95–1.18)	<0.001	<0.001
Temporal SUVR	1.43 (1.20–1.59)	1.10 (1.01–1.16)	<0.001	<0.001
Parietal SUVR	1.67 (1.13–1.84)	1.06 (0.98–1.12)	<0.001	<0.001
Occipital SUVR	1.47 (1.18–1.58)	1.15 (1.05–1.23)	<0.001	<0.001
^18^F-T807 PET				
Inferior temporal SUVR	1.25 (1.17–1.42)	1.08 (1.05–1.22)	<0.001	0.065
Braak I/II SUVR	1.00 (0.91–1.22)	0.92 (0.85–1.00)	0.009	0.213
Braak III/IV SUVR	1.12 (1.06–1.24)	1.02 (0.99–1.10)	<0.001	0.023
Braak V/VI SUVR	1.07 (0.99–1.16)	0.99 (0.91–1.04)	0.002	0.161

Values are median (interquartile range).

CAA, cerebral amyloid angiopathy; HTN-SVD, hypertensive small vessel disease.

Additionally, we performed a sensitivity analysis comparing patients with CAA to HTN-SVD who suffered deep haematoma (*n* = 14; 4 with strictly deep CMBs and 10 with lobar CMBs). The results retained similar trends, with significantly higher global and regional amyloid burdens (both *P* < 0.05) and a significantly higher tau burden in the inferior temporal lobe (age-adjusted *P* = 0.027) and Braak III/IV ROIs (age-adjusted *P* = 0.028) in CAA compared with HTN-SVD (Supplementary Table 1).

### cSS is associated with cerebral tau pathology in CAA

In order to clarify whether any relationships exist between tau pathology and CAA-related neuroimaging signatures in patients with CAA, we investigated the associations between the inferior temporal T807 SUVR and the following neuroimaging markers: whole cortex PiB SUVR, lobar CMB number, WMH volume, CSO-PVS grade, total cSS score and CAA score (Table 3). In univariable analysis, a higher whole cortex amyloid burden (*β* = 0.11, 95% CI 0.02–0.19), total cSS score (*β* = 0.11, 95% CI 0.05–0.18) and CAA score (*β* = 0.13, 95% CI 0.05–0.22) were significantly associated with a higher inferior temporal tau burden. The associations between inferior temporal tau and total cSS score or CAA score remained unchanged after adjustment for age and *ApoE4* status (both *P* < 0.05; Table 3, Model 2). We then built another quantile regression model that included whole cortex PiB SUVR as a covariate (Table 3, Model 3) to investigate the independent association between cSS score/CAA score and tau pathology. Both the total cSS score (*β* = 0.12, 95% CI 0.04–0.21) and CAA score (*β* = 0.12, 95% CI 0.03–0.21) were independently associated with the inferior temporal tau load. Similar results showing a significant correlation between the cerebral limbic region tau burden (Braak Stage III/IV ROI T807 SUVR) and the severity of cSS or CAA score were also observed in further analyses (Supplementary Table 2). Additionally, the association between inferior temporal tau burden and cSS score (*β* = 0.15, 95% CI 0.06–0.23) or CAA score (*β* = 0.12, 95% CI 0.03–0.20) remained unchanged if including cases of HTN-SVD/cSS(+) into the full model analyses as these patients may also harbour underlying CAA.

**Table 3:** Associations between inferior temporal tau and neuroimaging markers in CAA (expressed as increase per 1 standard deviation)

	Model 1 (Univariable)		Model 2 (Age- and ApoE4-adjusted)		Model 3 (Age-, ApoE4- and PiB SUVR-adjusted)	
Parameter	*β* (95% CI)	*P*-value	*β* (95% CI)	*P*-value	*β* (95% CI)	*P*-value
PiB whole cortex SUVR	0.11 (0.02–0.19)	0.02	0.10 (−0.02 to 0.01)	0.071	-	-
Lobar CMB number	0.06 (−0.16 to 0.14)	0.114	0.08 (0.00–0.17)	0.061	-	-
Total cSS score	0.11 (0.05–0.18)	0.002	0.13 (0.01–0.25)	0.040	0.12 (0.04–0.21)	0.006
WMH volume	0.06 (−0.07 to 0.19)	0.335	0.03 (−0.08 to 0.14)	0.557	-	-
CSO-PVS grade	0.04 (−0.06 to 0.15)	0.385	0.02 (−0.08 to 0.13)	0.644	-	-
CAA score	0.13 (0.05–0.22)	0.003	0.14 (0.05–0.22)	0.003	0.12 (0.03–0.21)	0.012

CMB, cerebral microbleeds; cSS, cortical superficial siderosis; CSO-EPVS, enlarged perivascular spaces in centrum semiovale; WMH, white matter hyperintensities.

The diagnostic accuracy of cSS and CAA score for predicting a positive tau PET scan was assessed using receiver operating characteristic curve analyses (Supplementary Fig. 1). Based on the Youden index, the optimal diagnostic cut-off was ≥2 for the total cSS score and ≥4 for the CAA score, respectively. The diagnostic performances of these MRI markers are presented in Table 4. The CAA score (AUC 0.825) was the best predictor of a positive tau PET scan, with a sensitivity of 76.9% (95% CI 46.2–95.0%), specificity of 77.9% (52.4–93.6%), positive predictive value of 71.3% (50.1–86.2%) and negative predictive value of 82.4% (62.7–92.9%). The total cSS score achieved similar diagnostic accuracy (AUC 0.812). The diagnostic performances of cSS and CAA scores in predicting positive tau scan remained similar if inclusion of cases of both CAA and HTN-SVD/cSS(+) (Supplementary Table 3).

**Table 4:** Diagnostic performance of cSS and CAA scores in predicting positive tau scan in CAA

	cSS(+)	Total cSS score	CAA score
Cut-off value	Presence	≥2	≥4
Sensitivity, %	69.2 (38.6–90.9)	69.2 (38.6–90.9)	76.9 (46.2–95.0)
Specificity, %	72.2 (46.5–90.3)	88.9 (65.3–96.6)	77.9 (52.4–93.6)
AUC	0.712 (0.516–0.898)	0.812 (0.646–0.978)	0.825 (0.679–0.970)
PPV, %	64.3 (44.0–80.5)	81.8 (53.7–94.6)	71.4 (50.1–86.2)
NPV, %	76.5 (57.8–88.5)	80.0 (63.5–90.2)	82.4 (62.7–92.9)

AUC, area under the curve; CAA, cerebral amyloid angiopathy; cSS, cortical superficial siderosis; NPV, negative predictive value; PPV, positive predictive value.

### Cerebral tau pathology is associated with neurodegeneration in CAA

**Figure 4:**
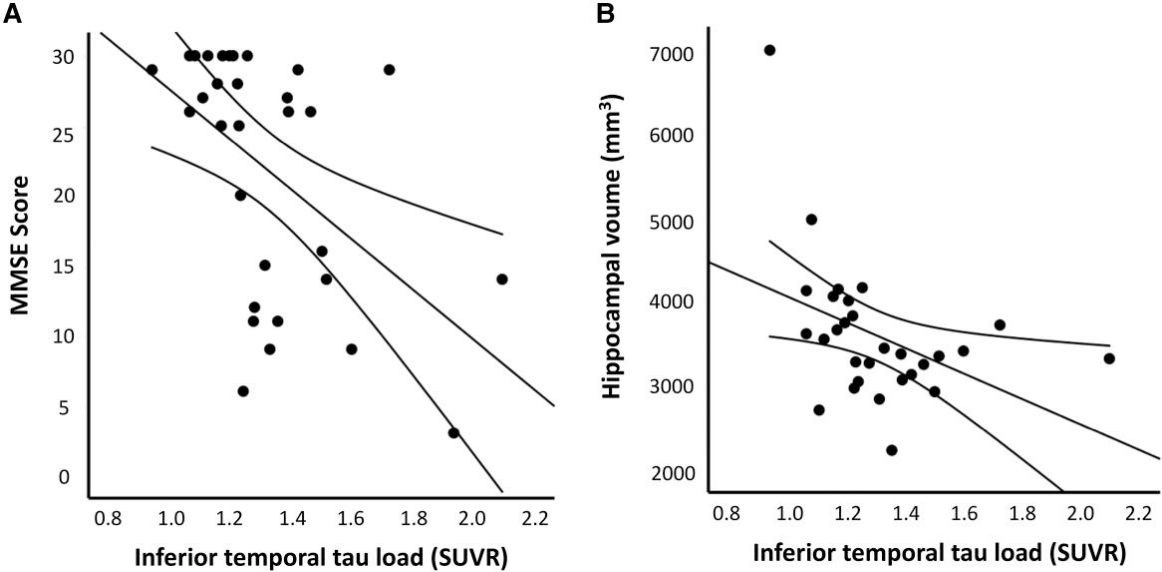
**Relationship between the inferior temporal tau burden and neurodegenerative markers in CAA.** A higher inferior temporal tau burden was significantly associated with (**A**) a lower MMSE score (Spearman’s *ρ* = −0.56, *P* = 0.001) and (**B**) smaller mean hippocampal volume (Spearman’s *ρ* = −0.49, *P* = 0.007).

To understand the clinical significance of tau pathology in CAA, we assessed the relationship between the inferior temporal T807 SUVR and global cognitive performance using the MMSE score as a surrogate of cognitive status. A higher inferior temporal T807 SUVR correlated significantly with a lower MMSE score (Spearman’s *ρ* = −0.56, *P* = 0.001; Fig. 4A). This association remained significant after adjustment for age, years of education and CAA score in the quantile regression model (*β* = −7.8, 95% CI −13.8 to−1.8; Supplementary Table 4). Consistent associations were demonstrated in similar regression models with adjustment for number lobar CMBs, CSO-PVS or WMH volume instead of CAA score (all *P* < 0.05; data not shown). On the other hand, no significant correlation was observed between the inferior temporal T807 SUVR and MMSE score in patients with HTN-SVD (Spearman’s *ρ* = 0.22, *P* = 0.273; data not shown).

We also investigated the relationship between tau pathology and neuroimaging markers of neurodegeneration, including hippocampal volume and mean cortical thickness in CAA. The inferior temporal T807 SUVR correlated significantly with a lower hippocampal volume (Spearman’s *ρ* = −0.49, *P* = 0.007; Fig. 4B) and mean cortical thickness (Spearman’s *ρ* = −0.40, *P* = 0.032). After adjustment for possible confounders, the association remained significant for hippocampal volume (*β* = −644.2, 95% CI −1241.0 to −47.5; Supplementary Table 4), but not for mean cortical thickness (*β* = −0.01, 95% CI −0.17 to 0.14; Supplementary Table 4).

## Discussion

This cross-sectional study of patients with SVD-related ICH who underwent both amyloid and tau scans reveals that patients with sporadic CAA exhibit higher amyloid and tau uptakes compared with patients with HTN-SVD. In CAA, tau accumulation was positively associated with the severity of vascular pathology, especially cSS. Additionally, the cerebral tau burden was independently associated with poorer global cognition and lower hippocampal volume. Overall, our findings indicate hyperphosphorylated tau protein accumulates in severe sporadic CAA and imply that the tau pathology causes neurodegeneration independently of vascular pathology.

CAA involves cerebrovascular deposition of Aβ, which is also the primary component of senile plaques in Alzheimer’s disease; therefore, these two diseases commonly overlap.^30,31^ Despite their frequent co-occurrence, the mechanisms that lead to brain injury in CAA and Alzheimer’s disease probably differ, in that vascular dysfunction appears to drive CAA-related brain injury while Aβ-triggered synapse and neuron loss and subsequent accumulation of hyperphosphorylated tau tangles are the main pathways that lead to neurodegeneration in Alzheimer’s disease.^2,32^ Tau pathology is traditionally not considered to be a prominent feature of CAA, although a small number of reports have observed tau deposition in hereditary or sporadic CAA.^33-36^ This report is the first larger study to investigate tau pathology in patients with sporadic CAA who presented with symptomatic ICH and confirms a higher tau burden in CAA compared with HTN-SVD. Additionally, we found higher tau deposition in regions that are related to Braak staging, suggesting that the tau accumulation in sporadic CAA probably follows a similar pattern as the patterns of tau deposition in Alzheimer’s disease.

One important observation of the current study is the association between cerebral tau pathology and vascular pathology in CAA, and more specifically, the relationship between tau pathology and the severity of cSS. This finding is in line with a previous study that showed the CSF p-tau level is more closely associated with cSS than lobar haematoma in CAA.^37^ Supporting evidence from case reports also indicates higher tau PET uptakes in cortical regions affected by cSS.^35,36^ Kim *et al.*^38^ studied patients with CAA with memory impairment and showed patients with cSS presented with more Alzheimer’s disease-related features, including a higher *APOE* ε4 frequency, more severe memory dysfunction and lower hippocampal volume. The same findings were observed in a meta-analysis of subjects with cognitive impairment, in that there is an association between the presence of cSS and Alzheimer’s disease.^39^ Currently, there is limited evidence from a mechanistic viewpoint to explain the relationship between cSS and cerebral tau accumulation. This observation could be suggestive of a possible relationship between leptomeningeal Aβ vasculopathy and cerebral tau pathologies, as cSS has been associated with a higher severity of CAA in the leptomeningeal vessels, but not in the cortical vessels.^40^ Additionally, pathological analyses have found tau-immunopositive neurites around amyloid-laden vessels,^7-9^ implying a potential interaction between CAA and perivascular accumulation of hyperphosphorylated tau.

An interesting observation of this study is the association between cerebral tau pathology and vascular markers, but not amyloid burden measured on PiB PET scans. One of the limitations of PiB PET is that this imaging technique cannot differentiate between vascular Aβ and parenchymal Aβ plaques; therefore, the PiB uptake signal in the patients in this study may represent the overall vascular and parenchymal Aβ burden. Another point to be considered is that vascular markers reflect the consequent haemorrhagic and/or ischaemic lesions from CAA and do not necessarily correlate with the severity of the vascular amyloid load.^41^ These two processes (amyloid deposition and vascular injuries) seem to develop in different stages of the disease, with the ischaemic and haemorrhagic injuries appearing at a later stage.^42^ As haemorrhagic lesions including cSS tend to develop at a later stage, the significant association between tau pathology and vascular markers rather than the amyloid load may imply that the tau pathology is more of an end-stage finding in sporadic CAA.

The presence of tau pathology appears to represent overlapping Alzheimer’s disease in subjects with CAA. Our study reveals that the tau burden in CAA correlates with a lower global cognitive score and smaller hippocampal volume, both of which are markers suggestive of Alzheimer’s disease-related neurodegeneration. These findings are in line with a previous study by Schoemaker *et al*.,^5^ who found that concomitant tau pathology in patients with CAA and cognitive impairment were more related to the amnestic phenotype. Similar patterns of tau accumulation in Alzheimer’s disease-susceptible regions were also observed in this study, suggesting that patients with concomitant tau deposition probably have a mixed cerebrovascular Aß and Alzheimer’s disease-related tau pathology. Additionally, findings from hereditary CAA, which represents a form of relatively pure CAA, further support the observation that hippocampal atrophy is not typically related to vascular Aß deposition.^4^ Thus, our findings and the work of others imply there is a frequent overlap with Alzheimer’s disease pathology in sporadic CAA. The presence of a low cognitive score (particularly in the memory domain), lower hippocampal volume or severe cSS could represent indicators of concomitant Alzheimer’s disease in sporadic CAA.

Our study has several limitations. First, we did not include age-matched healthy controls to demonstrate that tau pathology in sporadic CAA is more extensive than the effect of aging. The difference in tau burden between CAA and HTN-SVD became less significant after accounting for the effects of age. Although we adjusted for age in our statistical models, our results still need to be interpreted cautiously. Second, coexisting vascular pathology of hypertensive arteriosclerosis may be present in the CAA group, since the prevalence of chronic hypertension in the CAA group was high. However, there is no specific neuroimaging marker for HTN-SVD, except for the presence of deep bleeds. To address this risk of bias, we selected our CAA cases based on the MRI criteria of strictly lobar bleeds, but the possibility of concomitant arteriosclerosis and its contribution to tau pathology could not be assessed in our study. Third, we used ^18^F-T807 to assess tau pathology in the current study. This first-generation tau tracer has a high binding affinity for the paired helical filament of tau and also frequently shows off-target binding, including to haematoma (Fig. 2). We excluded the brain regions affected by haematoma from our PET quantitative analysis to minimize the confounding effects of off-target binding. Fourth, the cognitive function of our patients was determined using screening tools and the patients did not routinely undergo detailed neuropsychological assessments. Thus, we cannot evaluate whether changes in individual cognitive domains may be more specifically related to underlying tau pathology. As our participants were recruited from patients who had suffered a symptomatic ICH episode, their global cognitive scores may also potentially be influenced by the stroke event. In the current cohort, we did not exclude patients with cognitive dysfunction prior to enrolment, which may reflect pre-existing Alzheimer’s disease. Three (9.7%) patients in our CAA-ICH group already had an established clinical diagnosis of Alzheimer’s disease before the symptomatic ICH event. Investigation of biomarkers, especially Aß40, Aß42 and p-tau, in CSF may provide further insights; however, these data are unfortunately not available for our cohort. Lastly, the sample size in this study was relatively small, and our findings should be mainly considered to be exploratory. However, this is the largest amyloid and tau imaging study of a haemorrhagic SVD cohort reported to date, and we believe our results provide important insight into understanding the presentation of amyloid and tau pathology in sporadic CAA.

In conclusion, patients with sporadic CAA who presented with symptomatic ICH had a higher frequency of cerebral tau pathology in Alzheimer’s disease-signature regions, and tau accumulation was associated with the severity of CAA-related vascular pathology. The cerebral tau burden in CAA contributes to neurodegeneration in the hippocampus and a lower cognitive score, and this process is independent of the vascular pathology. The results of this study suggest that intensive effort is essential to characterize the entire Alzheimer’s disease-CAA spectrum in aging individuals and in the ICH population, in order to investigate how these two closely related—but independent amyloid pathologies—influence the clinical outcomes of patients.

## Supplementary Material

fcae086_Supplementary_Data

## Data Availability

All data from this article are being held within the National Taiwan University Hospital and will be shared with qualified investigators on request.
