# Supplementary material for: Cerebral tau pathology in cerebral amyloid angiopathy
Source: Brain Commun. 2024 Mar 12;6(2):fcae086. doi: 10.1093/braincomms/fcae086 (PMC11024817; doi:10.1093/braincomms/fcae086)
Supplement: fcae086_Supplementary_Data [file fcae086_Supplementary_Data.pdf]

Supplementary figure 1: The receiver operating characteristic (ROC) curves of vascular markers in predicting positive tau scans.

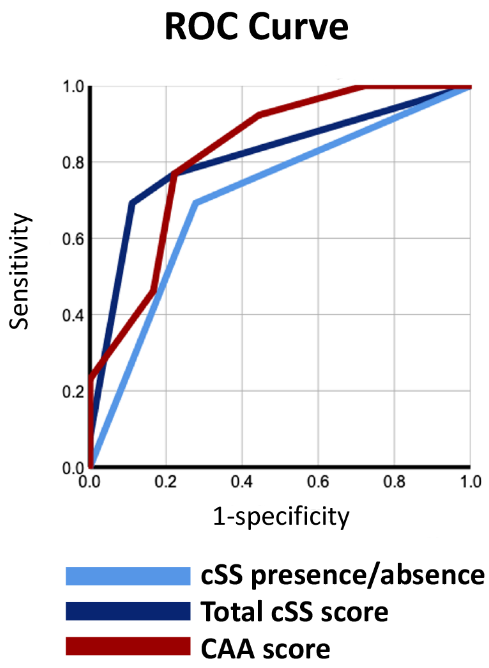

Supplementary Table 1 Comparison of amyloid and tau PET findings between CAA and HTN-SVD/Deep ICH.

Parameter	CAA (n=31)	HTN-SVD (n=14)	*P*-value	Age-adjusted *P*-value
11C-PiB PET				
Whole cortex SUVR	1.52 (1.11-1.65)	1.08 (1.00-1.14)	0.003	<0.001
Frontal SUVR	1.57 (1.09-1.66)	1.04 (0.95-1.18)	0.002	0.001
Temporal SUVR	1.43 (1.20-1.59)	1.10 (1.01-1.16)	<0.001	<0.001
Parietal SUVR	1.67 (1.13-1.84)	1.06 (0.98-1.12)	<0.001	0.001
Occipital SUVR	1.47 (1.18-1.58)	1.15 (1.05-1.23)	0.006	0.021
18F-T807 PET				
Inferior temporal SUVR	1.25 (1.17-1.42)	1.08 (1.05-1.22)	0.002	0.027
Braak I/II SUVR	1.00 (0.91-1.22)	0.92 (0.85-1.00)	0.186	0.300
Braak III/IV SUVR	1.12 (1.06-1.24)	1.02 (0.99-1.10)	0.002	0.028
Braak V/VI SUVR	1.07 (0.99-1.16)	0.99 (0.91-1.04)	0.021	0.124

Values are median (interquartile range). CAA: cerebral amyloid angiopathy; HTN-SVD: hypertensive small vessel disease; PiB: Pittsburgh compound B.

Supplementary Table 2 Correlation analysis between Braak stage III/IV tau and neuroimaging markers in CAA (expressed as an increase of per 1 standard deviation)

	**Model 1 (Univariable)**		**Model 2 (Age- and ApoE4- adjusted)**		**Model 3 (Age-, ApoE- and PiB SUVR-adjusted)**	
	β (95% CI)	***P* value**	β (95% CI)	**P value**	β (95% CI)	**P value**
PiB whole cortex SUVR	0.07 (0.00-0.14)	0.043	0.06 (-0.02-0.14)	0.116	-	-
Lobar CMB number	0.05 (-0.01-0.10)	0.092	0.06 (0.00-0.12)	0.050	-	-
Total cSS score	0.06 (0.00-0.13)	0.053	0.06 (-0.01-0.13)	0.113	0.08 (0.01-0.15)	0.019
WMH volume	0.00 (-0.08-0.08)	0.952	0.00 (-0.07-0.08)	0.962	-	-
CSO-PVS grade	0.06 (0.01-0.11)	0.025	0.04 (-0.01-0.09)	0.106	-	-
CAA score	0.06 (-0.01-0.13)	0.090	0.08 (0.01-0.15)	0.032	0.06 (0.00-0.13)	0.053

CAA: cerebral amyloid angiopathy; CMB: cerebral microbleeds; cSS: cortical superficial siderosis; CSO-EPVS: enlarged perivascular spaces in centrum semiovale; PiB: Pittsburgh compound B; SUVR: standardized uptake value ratio; WMH: white matter hyperintensities.

Supplementary Table 3. Diagnostic performance of cSS and CAA scores in predicting positive tau scan in CAA and HTN-SVD/cSS(+).

	**cSS(+)**	**Total cSS score**	**CAA score**
**Cutoff value**	Presence	≥2	≥4
**Sensitivity, %**	71.4 (41.9-91.6)	71.4 (41.9-91.6)	78.6 (49.2-95.3)
**Specificity, %**	61.9 (38.4-81.9)	85.7 (63.7-97.0)	81.0 (58.1-94.6)
**AUC**	0.667 (0.481-0.852)	0.782 (0.615-0.950)	0.833 (0.698-0.968)
**PPV, %**	55.6 (39.8-70.3)	76.9 (52.6-90.9)	73.3 (52.2-87.4)
**NPV, %**	76.5 (57.1-88.8)	81.8 (63.1-91.6)	85.0 (67.1-94.0)

AUC: Area under curve; CAA: Cerebral amyloid angiopathy; cSS: Cortical superficial siderosis; NPV: negative predictive value; PPV: positive predictive value; SUVR: Standardized uptake value ratio (relative to pons);

Supplementary Table 4 Quantile regression analysis between inferior temporal tau and neurodegeneration markers in CAA (expressed as an increase of per 1 standard deviation of SUVR)

**Dependent variable**	**β (95% CI)**	***P* value**
MMSE scores	-7.8 (-13.8 - -1.8)	0.013
Hippocampal volume	-644.2 (-1241.0 - -47.5)	0.036
Mean cortical thickness	-0.01 (-0.17-0.14)	0.874

Covariates: Age, educational years, CAA scores. Inferior temporal tau was standardized before entering as independent variable. CAA: cerebral amyloid angiopathy; MMSE: mini-mental status examination.
